# Zebrafish Primordial Germ Cell Migration

**DOI:** 10.3389/fcell.2021.684460

**Published:** 2021-06-23

**Authors:** Anne Aalto, Adan Olguin-Olguin, Erez Raz

**Affiliations:** Institute of Cell Biology, Center for Molecular Biology of Inflammation, University of Münster, Münster, Germany

**Keywords:** PGC, amoeboid migration, cell polarity, cell motility, chemotaxis, bleb, chemokine, Cxcr4

## Abstract

Similar to many other organisms, zebrafish primordial germ cells (PGCs) are specified at a location distinct from that of gonadal somatic cells. Guided by chemotactic cues, PGCs migrate through embryonic tissues toward the region where the gonad develops. In this process, PGCs employ a bleb-driven amoeboid migration mode, characterized by low adhesion and high actomyosin contractility, a strategy used by other migrating cells, such as leukocytes and certain types of cancer cells. The mechanisms underlying the motility and the directed migration of PGCs should be robust to ensure arrival at the target, thereby contributing to the fertility of the organism. These features make PGCs an excellent model for studying guided single-cell migration *in vivo*. In this review, we present recent findings regarding the establishment and maintenance of cell polarity that are essential for motility and discuss the mechanisms by which cell polarization and directed migration are controlled by chemical and physical cues.

## Introduction

Primordial germ cells (PGCs) are**** specified early in embryonic development, proceeding the specification of somatic gonadal cells, which occurs at a different position. Consequently, PGCs migrate within the embryo to reach the gonadal ridge, the site where they, with other cell types, form the gonad and differentiate into sperm and egg. In general, PGC migration may constitute an evolutionary mechanism for selecting the fittest cells (Cantú and Laird, 2017). It also offers a way to expand the options for animal body evolution, a feature termed “evolvability” (Kirschner and Gerhart, 1998; Payne and Wagner, 2019; Grimaldi and Raz, 2020).

In zebrafish embryos, PGCs are specified via the maternally inherited germ plasm, a phase-separated cytoplasmic structure containing specific RNA and protein determinants [e.g., *nanos* (*nos*), *vasa* (*vas*), and *dead end* (*dnd*)]. The germ plasm is initially enriched at four positions at the cleavage planes of the first two cell divisions and cells that inherit it develop into PGCs (Yoon et al., 1997); from these positions, directed by guidance cues the motile PGCs migrate toward their final target (Doitsidou et al., 2002; Knaut et al., 2003). During PGC migration, the germ plasm is found in perinuclear granules (Knaut et al., 2000; Weidinger et al., 2003).

Due to the unique characteristics of zebrafish embryos, namely their fast development and translucency, zebrafish PGCs constitute an attractive model for studying the migration process *in vivo*. Here, we present recent findings concerning the polarization and guided migration of zebrafish PGCs.

## PGC Motility and Directional Migration

Shortly following specification, zebrafish PGCs are round and immotile (Blaser et al., 2005); then, they start generating protrusions around the cell perimeter, before polarizing and becoming motile. Similar to *Dictyostelium discoideum*, melanoma cells and neutrophils, zebrafish PGCs employ a fast mode of motility termed “amoeboid migration” (Zatulovskiy et al., 2014; Hind et al., 2016; Song et al., 2020) that does not depend on specific adhesion to the substrate (Paluch et al., 2016). In contrast to mesenchymal cells (Ridley, 2011; Svitkina, 2018), the protrusions zebrafish PGCs produce are primarily blebs (Blaser et al., 2006; Olguin-Olguin et al., 2021), whose expansion is driven by hydrostatic pressure and cytoplasmic flow (Paluch and Raz, 2013; Goudarzi et al., 2019). Blebs emerge when the cell membrane detaches from the underlying actin cortex, producing a rounded protrusion pointing in the direction of cell migration (Paluch and Raz, 2013).

PGC migration involves the regulation of the expression level of E-cadherin, a cell-cell adhesion molecule (Bendel-Stenzel et al., 2000; Di Carlo and De Felici, 2000). Namely, the onset of PGC migration coincides with the downregulation of E-cadherin, whose expression level is controlled by Rgs14a, a scaffolding protein expressed in PGCs that integrates G protein and H-Ras/MAP kinase signaling pathways (Shu et al., 2010; Hartwig et al., 2014). Further, bleb formation and motility also require regulation of myosin-based contractility and cortex properties by the RNA binding protein Dnd (Goudarzi et al., 2012). Dnd controls the function of specific RNAs that play a role in PGC fate maintenance and motility acquisition (Goudarzi et al., 2012; Gross-Thebing et al., 2017). Specifically, Dnd was shown to decrease E-cadherin levels and promote bleb formation by reducing the level of the membrane-cortex linker Annexin5, thus freeing the membrane to inflate and form blebs. Last, Dnd may also promote contractility by protecting the RNA encoding myosin light chain kinase (MLCK) from microRNA-mediated degradation. Together, for their amoeboid movement PGCs require high contractility, flexible protrusion formation, and reduced adhesion levels.

As in several other vertebrates, such as mice (Molyneaux, 2003) and chickens (Stebler et al., 2004), PGC migration in zebrafish is guided by the chemokine Cxcl12a, which binds its receptor Cxcr4b on the cell surface (Doitsidou et al., 2002; Knaut et al., 2003; Molyneaux, 2003). Yet, polarized bleb formation and cell motility can occur also without chemokine signaling, and thus represent a primary behavioral feature of PGCs (Doitsidou et al., 2002; Gross-Thebing et al., 2020). Nevertheless, migrating PGCs do depend on Cxcl12a provided by cells located along the migration path to arrive at their specific intermediate and final targets (Doitsidou et al., 2002; Boldajipour et al., 2008).

## Polarized Distribution of Molecules and Organelles in Migrating PGCs

Motile cells should be polarized, such that a cell front and rear are defined (Ridley et al., 2003). For migrating PGCs, protrusions primarily form at the cell front (Doitsidou et al., 2002; Reichman-Fried et al., 2004; Olguin-Olguin et al., 2021), a location that also contains elevated levels of actin, positive regulators of actin polymerization, and myosin contraction activators (Kardash et al., 2010; Olguin-Olguin et al., 2021). The cell rear harbors molecules that enhance contractility as well, presumably to retract the cell rear during migration (Blaser et al., 2006).

Recently, a more comprehensive analysis revealed that other structures and molecules are unevenly distributed along the front–rear axis (Olguin-Olguin et al., 2021). Namely, the cell front contains actin structures termed actin brushes, whereas the cell rear is enriched for linker proteins that connect the plasma membrane to other structures within the cell. For example, similar to other cells types such as neutrophils and melanoma cells, the cell rear is enriched in Ezrin, a member of the ERM protein family (Lorentzen et al., 2011; Hind et al., 2016; Song et al., 2020), as well as Extended synaptotagmin-like protein 2a (Esyt2a) (Giordano et al., 2013) and Septins (Woods and Gladfelter, 2021). In its active form, Ezrin links the plasma membrane to the underlying actin cortex (Song et al., 2020), while Esyt2a connects the endoplasmic reticulum (ER) to the plasma membrane (Zaman et al., 2020), thereby locally inhibiting the inflation of blebs. The Septins localized to the cell rear form structures that increase cortex rigidity and serve as scaffolds for other proteins (Woods and Gladfelter, 2021). Similar findings were obtained in *Dictyostelium*, where talin, that links the membrane to the cortex, is enriched at the cell back (Tsujioka et al., 2012; Collier et al., 2017). In addition, the PGC rear also contains the microtubule organizing center (MTOC) and the Golgi apparatus (Olguin-Olguin et al., 2021), which is different from mesenchymal migrating cells, where these organelles are located toward the cell front (Yamada and Sixt, 2019). Interestingly, the ER is homogeneously distributed throughout the cell body but is not present within the front blebs, likely due to the actin brushes located at the bleb base (Olguin-Olguin et al., 2021). This pattern of ER localization could influence the protrusions’ biophysical properties, which may need to exclude the viscous organelle (Dayel et al., 1999).

The MLCK protein is found at the front of migrating PGCs, which could promote contractility and bleb formation at this aspect of the cell. Further, the cell front also harbors membrane invaginations that provide extra membrane for the expanding blebs (Goudarzi et al., 2017). Interestingly, these invaginations appear as the actin brushes form at the leading edge (Olguin-Olguin et al., 2021) and generate negative curvatures that have been shown to promote blebbing in other amoeboid migrating cells (Collier et al., 2017).

## The Cell Polarization Cascade

**FIGURE 1:**
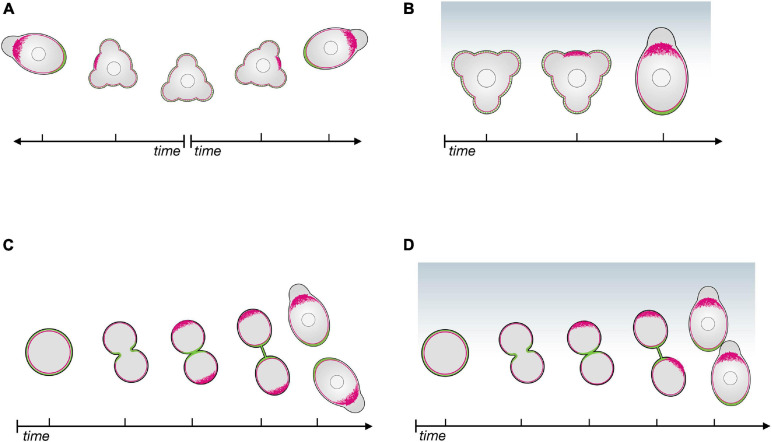
The polarity establishment cascade in zebrafish primordial germ cells in the absence of and in response to chemoattractant. **(A)** In the absence of chemoattractant, apolar PGCs initiate polarization by enhancing actin polymerization at a random position around the cell perimeter (red); proteins such as Ezrin and Esyt2a (green) accumulate at the opposite side of the cell, that becomes the cell rear. **(B)** In the presence of a chemokine gradient (Cxcl12a, blue), the cell front (actin in red) is established in the direction of the chemokine source, followed by the definition of the cell rear (green). **(C)** Upon cell division in the absence of chemoattractant, the linker molecules (Ezrin and Esyt2a in green) accumulate at the cleavage plane, defining the cell rear first; the cell front (actin in red) then forms at the opposite aspect of the cell. **(D)** When cell division occurs in the presence of a chemokine gradient (Cxcl12a, blue), the leading edge of both daughter cells form in the direction of the source of the chemoattractant.

The mechanisms that generate and maintain the cell’s front-rear axis can be investigated by monitoring the dynamic distribution of structures and molecules as the cell polarizes. For PGC migration, polarity can first be observed after cell specification (Hartwig et al., 2014) and after cell divisions (Olguin-Olguin et al., 2021). Importantly, during migration PGCs lose and re-gain polarity several times, as they alternate between apolar and polar phases (Reichman-Fried et al., 2004; Blaser et al., 2006; Olguin-Olguin et al., 2021). During their migration, PGCs are polar with respect to protrusion formation and move forward relative to neighboring cells. In the apolar phases, the cells send protrusions in all directions and do not migrate (Reichman-Fried et al., 2004). The loss of cell polarity allows the PGCs to introduce corrections in their migration path as they re-establish polarity according to the distribution of the guidance cue (Reichman-Fried et al., 2004; Minina et al., 2007). PGCs can also polarize without directional cues, but in such cases the newly established cell front is oriented in any direction (Reichman-Fried et al., 2004; Olguin-Olguin et al., 2021; Figure 1A). The transitions between polar and apolar states render cells especially sensitive and responsive to the chemoattractant gradient, as changes in migration direction are initially performed by apolar cells. Accordingly, when responding to a chemokine gradient, PGCs generate a leading edge in the direction of a chemoattractant source (Olguin-Olguin et al., 2021; Figure 1B).

The first visible indication of polarity is the formation of a filamentous actin-rich brush-like pattern at the future cell front (Figures 1A,B). This is preceded by activation of the small GTPase Rac1 and is followed by blebbing at this location. Importantly, inducing actin polymerization by light-controlled Rac1 activation at different locations around the PGC circumference causes the cell front to form at that position. Progressively, the linker proteins accumulate at the other end of the cell that develops into the cell rear (Figures 1A,B), suggesting that the forming cell front defines where the rear will be generated (Olguin-Olguin et al., 2021). Consistent with the idea that actin polymerization functions as a primary front determinant, inhibiting bleb formation (e.g., by downregulating contractility), does not abrogate the generation actin brushes (Olguin-Olguin et al., 2021), but, as discussed below, these are less stable.

Importantly, this polarization cascade and its kinetics are identical for both ligand-independent polarization and chemokine-directed PGC polarization (Figures 1A,B). These findings indicate that the main role of the chemokine Cxcl12a is to bias the underlying self-organizing polarity cascade toward regions with higher concentrations of the chemokine.

PGCs divide in average 3 times during their migration to the gonad region, and as mentioned above, the front-rear axis is also re-established after each cell division (Weidinger et al., 1999; Pfeiffer et al., 2018; Olguin-Olguin et al., 2021). During cytokinesis, Ezrin accumulates at PGCs’ cleavage furrow, similar to other cell types (McClatchey, 2014; Hiruma et al., 2017), where it likely functions in linking the membrane to the cytoskeleton. Interestingly, Esyt2a, another linker protein is enriched in the cleavage furrow as well. These two proteins are the first to exhibit a polar distribution at the exit from cell division (Olguin-Olguin et al., 2021; Figure 1C). Intriguingly, in this scenario the actin brushes and the cells’ front form at the part of the cell opposite from where the linker proteins are enriched (Olguin-Olguin et al., 2021; Figure 1C). These findings suggest that in this case, the cell rear directs the polarization cascade. When a chemokine gradient is present, the formation of the front in the daughter cells is biased in the direction of the source of the attractant, rather than being maintained at the site furthest from the cleavage plane (Olguin-Olguin et al., 2021; Figure 1D).

Interestingly, the localization of the MTOC to the rear of the cell is a late and temporally variable event in the PGC polarization cascade and appears to respond to the forming front-rear axis rather than directing its establishment. This contrasts with findings in several other cell types, in which during polarization the MTOC and the Golgi move ahead of the nucleus and play a role in forming and stabilizing the cell front [e.g., in wound healing (Bisel et al., 2008)].

In addition to the polarization cascades that can occur with or without chemoattractant, zebrafish PGCs can also be repelled from certain domains within the embryo after they arrive at the region where the gonad develops. At this stage, PGCs are kept away from nearby tissues that express lipid phosphate phosphatases, which, in turn, reduce the level of sphingosine-1-phosphate and lysophosphatidic acid around them (Paksa et al., 2016). Currently, the cellular and subcellular events relevant for the response of PGC to phospholipids have not been thoroughly studied; they represent an interesting future research direction.

## Generating and Maintaining Polarity

The mechanisms by which polarity is established and maintained can be elucidated by understanding the causal relationships and cross-dependency among the different polarized cellular components’ activities.

**FIGURE 2:**
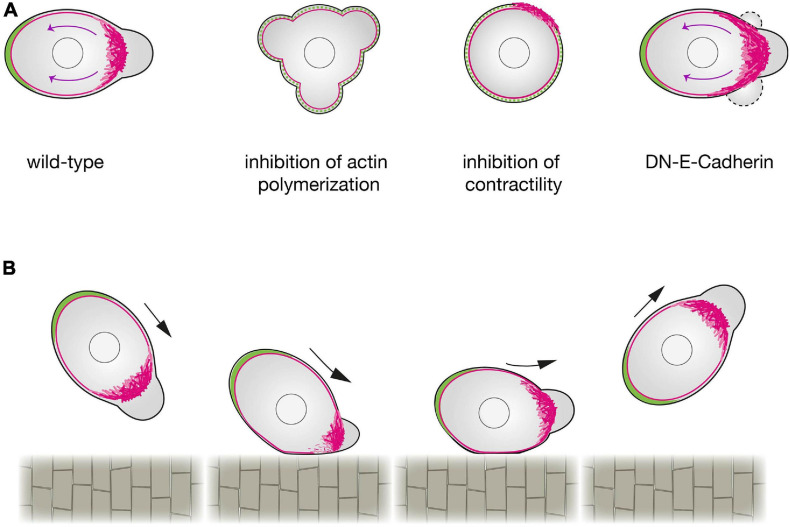
Regulation of zebrafish primordial germ cells polarization. **(A)** A schematic representation of a wild-type PGC (left) compared with a PGC in which actin polymerization (center left), contractility (center right) or cell-cell adhesion (right) are inhibited. Upon a reduction in actin polymerization (by inhibition of Rac1 activity), PGCs cannot form a cell front and polarity is not established. When contractility is blocked (e.g., by inhibition of ROCK activity), PGCs can generate actin brushes, but the cell rear is not defined. When E-Cadherin mediated cell-cell adhesion is inhibited, the actomyosin retrograde flow is enhanced, expanding the area of the cell front where blebs form. Retrograde actin flow is marked by magenta arrows. **(B)** The effect of contact with a barrier on PGC polarity. PGCs that make contact with the notochord at intermediate or steep angles exhibit a reflective behavior. A reduction in the actin brushes concentration at the side of the cell interacting with the barrier is observed, while the unaffected actin brushes at the opposite side define the new front that directs the migration away from the barrier.

Formation of blebs by actomyosin contraction is an important factor for defining the cell front, while, simultaneously, the flow of linker proteins away from the cell front is a key factor for defining the rear. This flow is the result of an actin assembly that pushes against the membrane, coupled with the contraction of myosin associated with the actin filaments (actin retrograde flow; Pollard et al., 2000). As in other cell types (Lin et al., 1996; Henson et al., 1999; Babich et al., 2012), inhibiting actin polymerization or myosin contractility in PGCs inhibits the retrograde flow (Grimaldi et al., 2020; Olguin-Olguin et al., 2021), which hinders the transport of the linker molecules Ezrin and Esyt2a as well as the MTOC and the Golgi to the back of the cell (Grimaldi et al., 2020; Olguin-Olguin et al., 2021; Figure 2A). Conversely, enhancing the rate of actin retrograde flow by increasing contractility results in strong polarization, in which a stable front protrusion and a cell rear form. Importantly, the retrograde flow of actin that functions in the initial steps of defining the cell rear is also sustained during the migration itself (Grimaldi et al., 2020; Olguin-Olguin et al., 2021), thereby maintaining the linker-depleted cell front and the linker-rich cell rear. A mathematical analysis of intracellular flows, showed that, shear stress may destabilize the binding of active Ezrin at the PGC front, favoring its accumulation at the cell rear (Dirks et al., 2021).

In addition to clearing the linkers, the actin brushes and the myosin contraction at the cell front also function to promote blebbing. Interestingly, the blebs themselves appear to stabilize the actin brushes, as evidenced by the finding that inhibiting blebbing activity reduces actin brush persistence (Olguin-Olguin et al., 2021). This finding uncovers an important positive feedback loop that, as described in other contexts, further stabilizes the cell front (Maiuri et al., 2015). Moreover, as the result of linker proteins accumulating at the cell rear, in this part of the cell blebbing is inhibited. These antagonistic relationships between the cell rear and front inhibit features of the front from spreading into the rear and vice versa. Accordingly, experimental overexpression of an activated version of the Ezrin protein interferes with the definition of the front, as bleb formation is inhibited (Olguin-Olguin et al., 2021).

This mutual inhibition between the cell front and the cell rear gives migrating PGCs a robust and stable polarity. This scenario of creating and maintaining cell polarization can be described theoretically by a modified version of the universal coupling of speed and persistence (UCSP) model (Maiuri et al., 2015; Olguin-Olguin et al., 2021). According to this model, the advection of the linker proteins to one side of the cell inhibits bleb formation at this location, while defining a bleb-prone area at the opposite side. PGC polarization occurs also at the end of cell division, when the cell rear develops first. As predicted by the model, in such a case where the cleavage plane is enriched in linker proteins, the bleb-prone area develops away from it.

An additional factor that contributes to defining the PGC front is E-cadherin-mediated cell-cell adhesion (van Roy and Berx, 2008; Grimaldi et al., 2020). Here, inhibiting E-cadherin function reduces persistent migration as a result of a less-focused cell front (Grimaldi et al., 2020). These findings suggest that E-cadherin, which binds actin at the cell interior and other E-cadherin molecules on neighboring cells, limits the extent of actin flow to the rear, thereby focusing the front and the bleb-prone area (Grimaldi et al., 2020; Figure 2A).

In summary, the process of repolarization following tumbling is initiated and oriented by actin polymerization at the future cell front, whereas at the end of cell division, the process of repolarization is controlled by the rear, which develops first. The antagonistic interactions between the front and the rear, coupled with the positive feedback between blebbing and actin polymerization and the function of cell-cell adhesion in restricting the spread of the front, lead to robust polarization of the migrating PGCs. The role of the guidance cues is thus merely to bias the position at which actin first accumulates, which initiates a self-maintaining polarization state oriented in the direction of the chemokine source.

## Physical Interaction of the Migrating PGCs With Embryonic Structures

Migrating cells physically interact with a broad range of tissues, extracellular matrix and other features in their environment during normal development and homeostasis [e.g., leukocytes and neural crest cells (Shellard and Mayor, 2019; Kameritsch and Renkawitz, 2020)], as well as in pathological conditions [e.g., cancer metastasis and inflammation (de Oliveira et al., 2016; Novikov et al., 2021)]. As zebrafish PGCs can migrate practically all over the embryo, especially in the absence of the guidance cue (Doitsidou et al., 2002; Gross-Thebing et al., 2020), they provide an excellent model for analyzing the behavior of migrating cells in different tissue contexts.

At the end of the first day of development, after PGCs arrive at their target, the guidance cues cease to be expressed at that location; however, PGCs remain motile (Paksa et al., 2016). Interestingly, the motile cells maintain the two bilateral cluster configurations, even without the chemoattractant, because of the presence of a physical barrier located at the midline of the embryo, the developing gut. Specifically, upon contacting the gut, the actin brushes at the leading edge are lost, and a new front is generated away from the barrier. This results in migration of PGCs away from the barrier at the midline, maintaining the two separated cell clusters. A qualitatively similar result has been observed when PGCs that lack the chemokine receptor reach barriers that they normally do not interact with (e.g., the notochord). In this case, the actin brushes at the leading edge are affected in a manner that depends on the angle of interaction, nonetheless causing the leading edge to form away from the barrier (Figure 2B).

In addition to being affected by the physical barriers within the embryo, the PGC migration path can also be influenced by the level of cell-cell adhesion in the environment. When PGCs encounter E-cadherin-depleted clusters of cells, their polarity is altered and they move away from the clone (Grimaldi et al., 2020). This behavior coincides with the results mentioned above concerning the stabilization of the cell front by E-cadherin; the change in polarity and migration direction can be explained by the asymmetrical actin flow at the leading edge. Specifically, the part of the PGC that interacts with the E-cadherin-depleted clone experiences faster retrograde flow, which effectively reduces actin concentration at this location. Under these conditions, the part of the leading edge that does not interact with the clone becomes dominant and directs the migration away from the E-cadherin-depleted area.

## Future Directions

In this review we highlight recent findings concerning the inherent polarization cascade within migrating PGCs and the robust mechanisms that maintain it once it formed. An interesting open question concerns the molecular and cellular mechanisms responsible for the disruption of the robust polarity at the end of each active migration phase and when PGCs stop migrating upon arrival at the gonad. Another open question relates to the precise mechanisms by which Cxcr4 activation promotes actin polymerization at the future front to initiate the formation of the front-rear axis, thereby orienting the otherwise stochastic self-propagated polarization cascade.

## Author Contributions

AA, AO-O, and ER wrote the manuscript. All authors contributed to the article and approved the submitted version.

## Conflict of Interest

The authors declare that the research was conducted in the absence of any commercial or financial relationships that could be construed as a potential conflict of interest.
